# FACTORS ASSOCIATED WITH DE-HOSPITALIZATION OF CHILDREN AND ADOLESCENTS WITH COMPLEX CHRONIC CONDITION

**DOI:** 10.1590/1984-0462/2021/39/2020118

**Published:** 2021-06-23

**Authors:** Michelli Christina Magalhães Novais, Deusiane Santos Victor, Danielle da Silva Rodrigues, Bruno Oliveira Freitas, Nilo Manoel Pereira Vieira Barreto, Daiana de Jesus da Silva Mendes, Micheli Bernardone Saquetto

**Affiliations:** a Centro Universitário Jorge Amado, Salvador, BA, Brazil.; b Hospital Martagão Gesteira, Salvador, BA, Brazil.; c Escola Bahiana de Medicina e Saúde Pública, Salvador, BA, Brazil.; d Universidade Federal da Bahia, Salvador, BA, Brazil.; e Faculdade Metropolitana de Camaçari, Camaçari, BA, Brazil.

**Keywords:** Child, Respiration, artificial, Patient discharge, Home nursing, Criança, Respiração artificial, Alta do paciente, Assistência domiciliar

## Abstract

**Objective:**

To assess the factors associated with the de-hospitalization of children and adolescents with complex chronic condition.

**Methods:**

This cross-sectional and retrospective study investigated a sample of children and adolescents admitted to the Dehospitalization Training Unit, from January 2012 to December 2017. Data were collected by consulting medical records and patient record books, from November 2018 to June 2019. The length of stay in the unit, de-hospitalization, readmissions, frequency and cause of death, age, sex, diagnosis, place of residence, number of caregivers and kinship, and use of devices were studied. The chi-square test was used to verify the association between the dependent variable (de-hospitalization) and the independent variables (age, sex, place of residence, use of devices, and clinical diagnosis).

**Results:**

A total of 93 patient records were analyzed, 37.6% aged between 7 months and 2 years old, 58.1% boys, 95.7% used tracheostomy, 92.5% gastrostomy, and 71% invasive mechanical ventilation. Hypoxic-ischemic encephalopathy was the diagnosis of 40.3% of the sample. Average hospitalization time was 288 ± 265 days; 60.2% were hospitalized between 31 days and one year, representing 50% of deaths. Of those de-hospitalized, 76.3% were discharged to the Ventilatory Assistance Homecare Program. De-hospitalization was associated with the child or adolescent's place of residence (p=0.027) and use of ventriculoperitoneal shunt (p=0.021).

**Conclusions:**

This study identified that de-hospitalization may be associated with the place of residence of the child or adolescent, with the highest number of discharges to the state capital, and non-dehospitalization when using ventricular-peritoneal shunt.

## RESUMO

**Objetivo:**

Avaliar os fatores associados à desospitalização de crianças e adolescentes com condição crônica complexa.

**Métodos:**

Estudo transversal e retrospectivo, que investigou a população de crianças e adolescentes internados na Unidade de Treinamento para Desospitalização (UTD), de janeiro de 2012 a dezembro de 2017. Os dados foram coletados por meio da consulta aos prontuários e livros de registros, de novembro de 2018 a junho de 2019. Foram estudados o período de internamento na UTD, a desospitalização, as reinternações, a frequência e causa dos óbitos, a idade, o sexo, o diagnóstico, o local de residência, o número de cuidadores e parentesco e o uso de dispositivos. Utilizou-se o teste do qui-quadrado para verificar a associação entre a variável dependente (desospitalização) e as variáveis independentes (idade, sexo, local de residência, uso de dispositivos e diagnóstico clínico).

**Resultados:**

O total de 93 prontuários de pacientes foi analisado, 37,6% tinham idade entre sete meses e dois anos, 58,1% eram meninos, 95,7% usavam traqueostomia, 92,5% gastrostomia e 71% ventilação mecânica invasiva. Encefalopatia hipóxico-isquêmica foi o diagnóstico de 40,3% da população. O tempo médio de hospitalização foi 288±265 dias; 60,2% ficaram internados entre 31 dias e um ano, representando 50% dos óbitos. Dos desospitalizados, 76,3% receberam alta para o Programa de Assistência Ventilatória Domiciliar (PAVD). A desospitalização foi associada ao local de procedência (p=0,027) e ao uso de derivação ventriculoperitoneal (DVP) (p=0,021).

**Conclusões:**

Identificou-se que a desospitalização esteve associada ao local de residência da criança ou adolescente e ao uso de DVP, sendo o maior número de altas para a capital do estado.

## INTRODUCTION

Complex chronic conditions (CCC) can be characterized by the presence of deficiency of function, dietary, drug, and technological input dependence, besides the need for multiprofessional care.^1^ ^,^ ^2^ Hospitalization incidence of children and adolescents with CCC in Brazil is 331 per 100 thousand inhabitants, which causes roughly 240 thousand hospitalizations every year, of which 13.5% require highly complex care.^3^ Among the etiologies that can lead to CCC, the three most frequent causes for hospitalizations are diseases of the respiratory system, neoplasms, and diseases of the nervous system.^3^

Children and adolescents with complex chronic diseases usually require the use of health services and technological support for long periods. They are often born and spend much of their childhood in pediatric hospitals.^4^ ^–^ ^6^ The reality of CCC patients raises questions about the effects of prolonged hospitalizations on a child's development and the impact on the quality of life of family members, who start to live in hospitals.^7^ In addition, the higher incidence of children with CCC increases the unavailability of intensive care beds for acute patients, generating high costs.^8^ In this context, as an alternative to long hospitalizations in intensive care units (ICU), many patients have been transferred to less complex units, such as those dependent on mechanical ventilation.^9^

The Dehospitalization Training Unit (*Unidade de Treinamento para Desospitalização* - UTD) can be characterized as a place of hospitalization for children with CCC, and aims to make their transition to their homes, in which they can be assisted by other programs, such as the Home Ventilatory Assistance Program (*Programa de Assistência Ventilatória Domiciliar* - PAVD). For this transition process to occur, family members and caregivers receive training and guidance. The entire teaching process takes place within the UTD and is carried out by the health professionals of the multidisciplinary team. Information provided to family members is essential, as different questions may arise during the treatment and monitoring of the child at home.^10^ ^,^ ^11^ Discharge to the PAVD is possible for patients who have clinical stability and those who have family members/caregivers able to provide basic home care.

The peculiarities of technological support, as well as the way of life of children and adolescents with CCC admitted to the UTD and their families, highlight the importance of exploring the characteristics of these patients and the factors that are associated with dehospitalization. The long stay in hospitals of children and adolescents who need technologies for survival is a current reality and few studies address this issue. Research on children and adolescents with CCC can provide support to promote action plans aimed at the real need of these patients. The presentation of results of treatment units for patients with CCC can collaborate with the performance strategies of other services and professionals who deal with this reality. Thus, the objective of this study was to analyze the factors associated with the dehospitalization of children and adolescents with CCC.

## METHOD

This is a cross-sectional and retrospective study that evaluated information from the medical records of children and adolescents admitted to the UTD of the philanthropic hospital Martagão Gesteira, in Salvador City, Bahia State, Brazil, from the creation of the unit, on May 25, 2012, until December 2017. UTD de-hospitalizes children and adolescents with CCC and those dependent on technologies for survival, such as mechanical ventilatory support. The sector receives patients with long hospital stays, who are clinically stable, but who, because of the underlying disease, use technological devices and need specific assistance.

The present study followed the recommendations of the Resolution of the National Health Council (CNS) No. 466, of December 12, 2012, to carry out research with human beings, and was approved by the Research Ethics Committee of Maternidade Climério de Oliveira, under opinion No. 3.024.335.

All medical records of children and adolescents (according to the classification of the Brazilian Institute of Geography and Statistics, which considers this population to be in the age group from zero to 18 years old), were admitted to the sector during the analyzed period. Data were collected by consulting patient records. Medical records of children with incomplete information were excluded from the study.

For the description of the sociodemographic and clinical characteristics of children and adolescents, a data collection instrument was built, which contained information regarding age, sex, diagnosis, place of residence (capital, metropolitan region, or inner part of the state), number of caregivers, kinship of caregivers, use of devices (tracheostomy, mechanical ventilation, gastrostomy, colostomy, VP shunt) during the period of stay in the UTD. The total period of hospitalization in the sector and dehospitalization (transfer to the PAVD, transfer to another home care program, or hospital discharge without the need for devices), the number of readmissions, and the cause of death were also investigated.

Data were stored and analyzed with the Statistical Package for the Social Sciences (SPSS) software, version 20.0, with the quantitative variables presented in measures of central tendency and dispersion, and the categorical variables in absolute and relative frequency. The chi-square test was used to verify the association between the independent variables (age, sex, clinical diagnosis, place of residence, use of devices) and the dependent variable (dehospitalization). A 5% significance level was established.

## RESULTS

When analyzing the medical records, a total of 94 children and adolescents were identified for being admitted to the UTD between May 25, 2012 and December 2017. Of these patients, 93 were included in the study and one was excluded due to lack of information relevant to research in medical records.

Most of the children and adolescents studied were aged between seven months and two years old, 58.1% (54/93) were boys, 95.7% (89/93) used tracheostomy, 92.5% (86/93) used gastrostomy, and 71% (66/93) used mechanical ventilation. As for the primary clinical diagnosis, 55.9% (52/93) of patients had alterations in their central nervous system, of which 40.4% (21/52) had specifically hypoxic-ischemic encephalopathy. Rare diseases, such as Krabbe's disease, Moebius syndrome, and choanal atresia, represent 11.8% (11/93) of diagnoses (Table 1).

**Table 1:** Sociodemographic and clinical characteristics of children who were admitted to the Dehospitalization Training Unit, from May 2012 to December 2017.

Characteristics		Total n=93 (%)
Age		
	0–6 months	25 (26.9)
	>6 months–2 years	35 (37.6)
	>2 years	33 (35.5)
	Mean±SD of age (months)	32±42
Sex		
	Girl	39 (41.9)
	Boy	54 (58.1)
Place of origin		
	Salvador City	39 (41.9)
	Metropolitan region	7 (7.5)
	Inner part of the state	47 (50.5)
Kinship of caregivers		
	Mother	42 (45.2)
	Mother/father	33 (35.5)
	Mother/grandmother	5 (5.4)
	Other family settings	13 (13.9)
	Mean±SD of caregivers numbers	2.08±0.78
Use of devices		
	Tracheostomy	89 (95.7)
	MV	66 (71.0)
	Gastrostomy	86 (92.5)
	Colostomy	2 (2.2)
	VP shunt	7 (7.5)
Diagnostic		
	Rare condition	11 (11.8)
	CNS alteration	52 (55.9)
	PNS alteration	17 (18.3)
	Congenic heart disease	4 (4.3)
	Congenital heart disease and CNS alteration	7 (7.5)
	Others	11 (11.8)
	Mean±SD of the hospitalization time (days)	288±265
	Mean±SD of re-hospitalization	1.56 ±2.45

MV: mechanical ventilation; VP shunt: ventriculoperitoneal shunt; CNS: central nervous system; PNS: peripheral nervous system; SD: standard deviation.

The hospitalization time was, on average, 288 ± 265 days, and 60.2% (56/93) of the children were hospitalized between 31 days to one year. The mean rehospitalization of patients was 1.56 ± 2.45 (Table 1).

A total of 58 children and/or adolescents were discharged, of which 75.9% (44/58) were discharged for PAVD, and 24.1% (14/58) for other home care programs, whereas 14 children and/or adolescents remained hospitalized in the UTD. For these 72 children and/or adolescents, an association was found between dehospitalization and the place of residence/origin (p=0.027) and the use of VP shunt (p=0.021) (Table 2).

**Table 2:** Factors associated with the dehospitalization of children with complex chronic conditions hospitalized at the Dehospitalization Training Unit, from May 2012 to December 2017.

Characteristics		Dehospitalization n=58 (%)	Time in UTD n=14 (%)	Total n=72 (%)	p-value
Age					0.430
	0–6 months	17 (29.3)	2 (14.3)	19 (26.5)	
	>6 months–2 years	21 (36.2)	5 (35.7)	26 (36.0)	
	>2 years	20 (34.5)	7 (50.0)	27 (37.5)	
Sex					0.431
	Girl	25 (43.1)	7 (50.0)	32 (44.4)	
	Boy	33 (56.9)	7 (50.0)	40 (55.6)	
Place of origin					0.027*
	Salvador City	28 (48.3)	3 (21.4)	31 (43.0)	
	Metropolitan region	7 (12.0)	0 (0.0)	7 (9.8)	
	Inner part of the state	23 (39.7)	11 (78.6)	34 (47.2)	
Use of devices					
	Tracheostomy	55 (94.8)	13 (92.8)	68 (94.4)	0.588
	MV	37 (63.8)	9 (64.3)	46 (63.9)	0.973
	Gastrostomy	53 (91.3)	12 (85.7)	65 (90.2)	0.411
	Colostomy	2 (3.4)	0 (0.0)	2 (2.8)	0.647
	VP shunt	1 (1.7)	3 (21.4)	4 (5.5)	0.021*
Diagnosis					0.619
	Rare condition	5 (8.6)	1 (7.1)	6 (8.3)	
	CNS alteration	30 (51.7)	11 (78.6)	41 (56.9)	
	PNS alteration	11 (18.9)	1 (7.1)	12 (16.6)	
	Congenic cardiopatics	7 (12.1)	0 (0.0)	7 (9.7)	
	Congenital heart disease and CNS alteration	5 (8.6)	1 (7.1)	6 (8.3)	
	Others	2 (3.4)	0 (0.0)	2 (2.8)	

MV: mechanical ventilation; VP shunt: ventriculoperitoneal shunt; CNS: central nervous system; PNS: peripheral nervous system; RS: respiratory system. Statistically significant difference with the chi-square test.

Of 93 children analyzed, 22.6% (21/93) died during the study period. The most frequent cause of death was sepsis, representing 52.4% (11/21), followed by 23.8% (5/21) of complications from the underlying disease. Among deaths, 47.6% (10/21) were of children who remained hospitalized for a period ranging from 31 days to one year.

## DISCUSSION

According to the results found, half of the hospitalized patients came from the inner part of the state, representing the vast majority of those who remained hospitalized and those who died. Almost all patients had tracheostomy and gastrostomy, and most were mechanically ventilated. The average period of hospitalization was long, and half of the patients who died had sepsis as the main cause of death.

Nervous system diseases are considered one of the main causes of prolonged hospitalization, with higher rates in the first year of life.^3^ The population of this study consisted of a large number of children with alterations in the central nervous system, most of whom were specifically diagnosed with hypoxic-ischemic encephalopathy. Therefore, different from the study by Hanashiro et al.,^9^ carried out in a similar sector, in which this syndrome represented the second largest diagnosis in the sample. Hypoxic-ischemic encephalopathy is a disorder of 26 per thousand live births incidence in underdeveloped countries.^12^ Babies with hypoxic-ischemic encephalopathy can develop cerebral palsy, the prognosis of early death is low, but there is an association with the development of long-term neurological sequelae.^13^ ^,^ ^14^ In addition, it is considered one of the main causes of disability in newborns.^15^

According to Hassani et al.,^16^ there is a high occupancy of pediatric ICU beds, resulting in high hospital costs. The study by Hanashiro et al.,^9^ carried out in sectors with proposals similar to those of UTD and PAVD, indicates that the transfer of 22 ICU patients to a mechanical ventilation dependent unit provided 8,643 bed-days for new hospitalizations in the pediatric ICU, corresponding to 98 bed-days per month. The same study revealed that the transfer of eight patients to mechanical ventilation at home made 4,022 bed-days available in the mechanical ventilation dependent unit in four years.^9^ Fortunately, the emergence of legal support for the provision of home mechanical ventilation to patients with chronic respiratory failure in 2018, via a single health system,^17^ made the transfer of dependents on mechanical ventilation to their homes more feasible.

The average length of hospital stay was 288 days, with 60.2% of children and adolescents hospitalized for between 31 days and one year, a period much longer than that of the study by Pinto et al.,^18^ who found an average of 35 days of stay hospital. Prolonged periods of hospitalization generate harmful consequences for the families involved,^7^ besides limiting the execution of activities and restricting the social participation of children with CCC, which can affect their motor and cognitive development.

There was an association between the use of VP shunt and the outcome of hospitalization. Children with chronic hospitalized conditions had a higher rate of infections,^19^ which may be related to the long period of time they remained hospitalized.^20^ In addition to prolonged hospitalization,^21^ the risk of infection increases with the use of invasive devices, such as VP shunt.^22^ In our study, the main cause of death was sepsis, and we were unable to establish the source of infection. Furthermore, the use of VP shunt may also be associated with complications of a mechanical nature, such as valve malfunction,^22^ which has the potential to increase intracranial pressure and cause disorders in the central nervous system,^23^ worsening the prognosis and increasing the time of hospitalization of children and adolescents with CCC.

All children from the metropolitan region were discharged. We highlight that PAVD assists only Salvador and neighboring cities, a situation that makes it more difficult to dehospitalize patients coming from the inner part of the state who, in their majority, do not have a residence in the capital or metropolitan region. Besides that, few cities in the inner parte of the state have the necessary structure and trained staff to offer adequate support to these children and adolescents at home.

For children with CCC to be dehospitalized, they not only need to be clinically stable, but their residence that will receive them must have the proper structure, with adequate hygiene and temperature conditions, as well as feasibility of access to care for urgency.^24^ In order to acquire this structure, financial resources are often unavailable for the relatives of philanthropic hospital patients. Thus, dehospitalization must be considered beyond the hospital context, encompassing social improvements that make the treatment of children with CCC at home viable, and the proposition of public policies directed to this end must be appropriate.

The American Thoracic Society has developed evidence-based guidelines for clinical practice and for the management of children using invasive mechanical ventilation at home. Children's stability for dehospitalization, the guarantee of medical care, and the safety of their homes and their community environment were criteria presented in the guidelines that can help providing better assistance to these children at home. Another criterion presented was family preparation, with the suggestion that at least two family caregivers be trained to care for the child dependent on invasive mechanical ventilation at home.^24^

For the safety of care for children with CCC at home, the family member/caregiver must know basic procedures, as well as be able to identify signs of clinical instability. Family members of UTD patients are trained to do so. In addition, they are subjected to supervised simulation, referring to the care that will be offered in the domestic environment, with performance in this period being decisive for the release for de-hospitalization. However, this preparation process is not always successful. Thus, insufficient understanding and ability to perform procedures necessary to care for children with CCC at home are important factors for prolonging hospitalization. However, this association was not investigated in the present study.

More than half of the children at UTD had only one caregiver, who was their parent. The dedication of parents to their children with chronic diseases restricts the time they have avialable for other activities, commonly impairing the permanence or the possibility of finding a job.^25^ Although the Brazilian government provides financial benefits for these children, Tavares et al.^26^ state that the strategies and actions promoted by public policies for children with CCC in Brazil are still primary and show signs of limited funding.

In addition to labor difficulties, associated family issues, such as those related to the care of the rest of the family, can stress parents.^25^ Therefore, Moreira et al.^4^ recommended the adoption of support initiatives for the family network, promoted even during the hospitalization process.

The present study has as a limitation the absence of longitudinal and prospective follow-up to investigate, for example, the hospital outcome of participants who remained hospitalized in the sector, the time of using mechanical ventilation, in addition to monitoring those who were discharged, gathering information such as influence of dehospitalization on the quality of life not only of these patients, but also of their families. Further studies should be carried out to monitor children and adolescents with CCC, not only in the hospital environment, but also at home, following their clinical and psychosocial evolution.

Present research made it possible to know the profile of children and adolescents with CCC in the only sector in Bahia State which is specialized in training for de-hospitalization in pediatrics. Dehospitalization was identified as associated with the patients’ place of residence and the use of VP shunt, with the highest number of discharges in the state capital.

Information in this study can help and promote the direction and elaboration of changes in public health policies, which ensure comprehensive and humanized care to children and adolescents with CCC and their families, emphasizing the need to create new units, such as UTD, and to provide social protection to dehospitalization.

## References

[1] Cohen E, Kuo DZ, Agrawal R, Berry JG, Bhagat SK, Simon TD (2011). Children with medical complexity: An emerging population for clinical and research initiatives. Pediatrics.

[2] Floriani CA (2010). Home-based palliative care: Challenges in the care of technology-dependent children. J Pediatr (Rio J).

[3] Moura EC, Moreira MC, Menezes LA, Ferreira IA, Gomes R (2017). Complex chronic conditions in children and adolescents: hospitalizations in Brazil, 2013. Cienc Saude Coletiva.

[4] Moreira MC, Albernaz LV, Sá MR, Correia RF, Tanabe RF (2017). Guidelines for a line of care for children and adolescents with complex chronic health conditions. Cad Saude Publica.

[5] Okido AC, Pizzignacco TM, Furtado MC, Lima RA (2012). Technology-dependent children: the maternal care experience. Rev Esc Enferm USP.

[6] Wise PH (2007). The future pediatrician: the challenge of chronic illness. J Pediatr.

[7] Dybwik K, Tollåli T, Nielsen EW, Brinchmann BS (2011). Fighting the system: families caring for ventilator-dependent children and adults with complex health care needs at home. BMC Health Serv Res.

[8] Edwards EA, Nixon GM, Australasian Paediatric Respiratory Group Working Party on Home Ventilation (2013). Paediatric home ventilatory support: changing milieu, proactive solutions. J Paediatr Child Health.

[9] Hanashiro M, Franco AO, Ferraro AA, Troster EJ (2011). Care alternatives for pediatric chronic mechanical ventilation. J Pediatr (Rio J).

[10] Góes FG, Cabral IE (2017). Discourses on discharge care for children with special healthcare needs. Rev Bras Enferm.

[11] Esteves JS, Silva LF, Conceição DS, Paiva ED (2015). Families’ concerns about the care of children with technology-dependent special health care needs. Investig Educ Enferm.

[12] Kurinczuk JJ, White-Koning M, Badawi N (2010). Epidemiology of neonatal encephalopathy and hypoxic-ischaemic encephalopathy. Early Hum Dev.

[13] Thom T, Haase N, Rosamond W, Howard VJ, Rumsfeld J, Manolio T (2006). Heart disease and stroke statistics - 2006 update: a report from the American Heart Association Statistics Committee and Stroke Statistics Subcommittee. Circulation.

[14] Jacobs SE, Berg M, Hunt R, Tarnow-Mordi WO, Inder TE, Davis PG (2013). Cooling for newborns with hypoxic ischaemic encephalopathy. Cochrane Database Syst Rev. 2013.

[15] Douglas-Escobar M, Weiss MD (2015). Hypoxic-ischemic encephalopathy a review for the clinician. JAMA Pediatr.

[16] Hassani SA, Navaei S, Shirzadi R, Rafiemanesh H, Masiha F, Keivanfar M (2019). Cost-effectiveness of home mechanical ventilation in children living in a developing country. Anaesthesiol Intensive Ther.

[17] Brazil - Ministério da Saúde Portaria n° 68, de 23 de novembro de 2018 (2018). Torna pública a decisão de incorporar a ventilação mecânica incvasiva domiciliar para insuficiência respiratória crônica, mediante pactuação tripartite no âmbito do Sistema Único de Saúde - SUS.

[18] Pinto M, Gomes R, Tanabe RF, Costa AC, Moreira MC (2019). Analysis of the cost of care for children and adolescents with medical complex chronic conditions. Cienc Saude Coletiva.

[19] de Carvalho AJ, Ferreira HM, Borges EF, Borges LH, Paula AL, Hattori WT (2019). Analyses of the effectiveness of a Brazilian pediatric home care service: a preliminary study. BMC Health Serv Res.

[20] Lisboa T, Faria M, Hoher JA, Borges LA, Gómez J, Schifelbain L (2007). The Prevalence of nosocomial infection in Intensive Care Units in the State of Rio Grande do Sul. Rev Bras Ter Intensiva.

[21] Leoncio JM, Almeida VF, Capobiango JD, Kerbauy G, Teresa M, Mendes G (2019). Impact of healthcare-associated infections on the hospitalization costs of children. Rev Esc Enferm USP.

[22] Jucá CE, Neto AL, Oliveira RS, Machado HR (2002). Treatment of hydrocephalus by ventriculoperitoneal Shunt: analysis of 150 consecutive cases in the Hospital of the Faculty of Medicine of Ribeirão Preto. Acta Cir Bras.

[23] Gusmão S, Silveira RL, Cabral G, Arantes A (2000). Clinical applications of hydrodinamics in ventriculoperitoneal shunt. Arq Bras Neurocir.

[24] Moore PE, Boyer D, O’Connor MG, Baker CD, Rettig JS, Sterni L (2016). Pediatric chronic home invasive ventilation. Ann Am Thorac Soc.

[25] Kish AM, Newcombe PA, Haslam DM (2018). Working and caring for a child with chronic illness: a review of current literature. Child Care Health Dev.

[26] Tavares TS, Duarte ED, Sena RR (2017). Social rights of children with chronic conditions: a critical analysis of Brazilian public policies. Esc Anna Nery.

